# Influence of O^6^-benzylguanine on the anti-tumour activity and normal tissue toxicity of 1,3-bis(2-chloroethyl)-1-nitrosourea and molecular combinations of 5-fluorouracil and 2-chloroethyl-1-nitrosourea in mice

**DOI:** 10.1038/sj.bjc.6690215

**Published:** 1999-03

**Authors:** M C Bibby, M J Thompson, J A Rafferty, G P Margison, R S McElhinney

**Affiliations:** Clinical Oncology Unit, University of Bradford, Bradford, BD7 1DP, UK; Department of Biomedical Sciences, University of Bradford, Bradford, BD7 1DP, UK; CRC Section of Genome Damage and Repair, Paterson Institute for Cancer Research,Christie Hospital NHS Trust, Manchester, M20 4BX, UK; University Chemical Laboratory, Trinity College, Dublin 2, Ireland

**Keywords:** O^6^-benzylguanine, nitrosoureas, anti-tumour, tissue toxicity, mouse

## Abstract

Previous studies have demonstrated that novel molecular combinations of 5-fluorouracil (5FU) and 2-chloroethyl-1-nitrosourea (CNU) have good preclinical activity and may exert less myelotoxicity than the clinically used nitrosoureas such as 1,3-bis(2-chloroethyl)-1-nitrosourea (BCNU). This study examined the effect of O^6^-alkylguanine-DNA-alkyltransferase (ATase) depletion by the pseudosubstrate O^6^-benzylguanine (BG) on the anti-tumour activity and normal tissue toxicity in mice of three such molecular combinations, in comparison with BCNU. When used as single agents at their maximum tolerated dose, all three novel compounds produced a significant growth retardation of BCNU-resistant murine colon and human breast xenografts. This in vivo anti-tumour effect was potentiated by BG, but was accompanied by severe myelotoxicity as judged by spleen colony forming assays. However, while tumour resistance to BCNU was overcome using BG, this was at the expense of enhanced bone marrow, gut and liver toxicity. Therefore, although this ATase-depletion approach resulted in improved anti-tumour activity for all three 5-FU:CNU molecular combinations, the potentiated toxicities in already dose-limiting tissues indicate that these types of agents offer no therapeutic advantage over BCNU when they are used together with BG. © 1999 Cancer Research Campaign


*British Journal of Cancer* (1999) **79**(9/10), 1332–1339
© 1999 Cancer Research Campaign
Article no. bjoc.1998.0215

### Influence of O⁶-benzylguanine on the anti-tumour activity and normal tissue toxicity of 1,3-bis(2-chloroethyl)-1-nitrosourea and molecular combinations of 5-fluorouracil and 2-chloroethyl-1-nitrosourea in mice

**MC Bibby[1], MJ Thompson[1,2], JA Rafferty[3], GP Margison[3] and RS McElhinney[4]**

[1]Clinical Oncology Unit and [2]Department of Biomedical Sciences, University of Bradford, Bradford, BD7 1DP, UK; [3]CRC Section of Genome Damage and Repair, Paterson Institute for Cancer Research, Christie Hospital NHS Trust, Manchester, M20 4BX, UK; [4]University Chemical Laboratory, Trinity College, Dublin 2, Ireland

**Summary** Previous studies have demonstrated that novel molecular combinations of 5-fluorouracil (5FU) and 2-chloroethyl-1-nitrosourea (CNU) have good preclinical activity and may exert less myelotoxicity than the clinically used nitrosoureas such as 1,3-bis(2-chloroethyl)-1-nitrosourea (BCNU). This study examined the effect of $O^6$-alkylguanine-DNA-alkyltransferase (ATase) depletion by the pseudosubstrate $O^6$-benzylguanine (BG) on the anti-tumour activity and normal tissue toxicity in mice of three such molecular combinations, in comparison with BCNU. When used as single agents at their maximum tolerated dose, all three novel compounds produced a significant growth retardation of BCNU-resistant murine colon and human breast xenografts. This in vivo anti-tumour effect was potentiated by BG, but was accompanied by severe myelotoxicity as judged by spleen colony forming assays. However, while tumour resistance to BCNU was overcome using BG, this was at the expense of enhanced bone marrow, gut and liver toxicity. Therefore, although this ATase-depletion approach resulted in improved anti-tumour activity for all three 5-FU:CNU molecular combinations, the potentiated toxicities in already dose-limiting tissues indicate that these types of agents offer no therapeutic advantage over BCNU when they are used together with BG.

**Keywords:** $O^6$-benzylguanine, nitrosoureas, anti-tumour, tissue toxicity, mouse

The cytotoxic action of the chloroethylnitrosoureas and related methylating agents ($O^6$-alkylating agents) has long been exploited in the treatment of cancer. Their cytotoxicity, mutagenicity, carcinogenicity and clastogenicity has been shown to be due in large part to their ability to alkylate DNA and in particular to form $O^6$-alkylguanine ($O^6$-alkG). Cells, both normal and neoplastic, possess the ability to repair this adduct via a single step reaction involving $O^6$-alkylguanine-DNA alkyltransferase (ATase) (Yarosh, 1985; Pegg and Dolan, 1987; Margison and O'Connor, 1990; Pegg, 1990; Pegg and Byers, 1992). ATase catalyses the transfer of the alkyl group from DNA to a cysteine acceptor site within the protein, resulting in the auto-inactivation of the ATase. Restoration of activity therefore requires de novo protein synthesis and so the capacity of cells to repair $O^6$-alkG is transiently limited by the number of ATase molecules present. Consequently, the sensitivity of a given normal or neoplastic cell to the biological effects of $O^6$-alkG strongly correlates with ATase activity (Brennand and Margison, 1986; Jelinek et al, 1988; Kaina et al, 1991; von Hofe et al, 1992; Brent et al, 1993; Dumenco et al, 1993; Nakatsuru et al, 1993; Jelínek et al, 1996; Moritz et al, 1995). For this reason the depletion of ATase in tumours has become a therapeutic target in order to sensitize tumours to $O^6$-alkylating agent treatment (Dolan et al, 1985; Gerson et al, 1988; Mitchell et al, 1992; Wedge and Newlands, 1992*b*; Margison et al, 1996; Kurpad et al, 1997).

One agent that can be used to deplete cellular ATase levels is $O^6$-benzylguanine (BG) (Bronstein et al, 1992; Dolan et al, 1994; Dolan and Pegg, 1997). Inactivation of ATase with BG has been demonstrated to be irreversible, indicating that it acts as an alternative substrate for the protein (Dolan et al, 1990; Pegg et al, 1993) and can potentiate the effects of anti-tumour agents against otherwise resistant cells and tumours (Mitchell et al, 1992; Baer et al, 1993; Kurpad et al, 1997). The combination of BG and 1,3-bis(2-chloroethyl)-1-nitrosourea (BCNU) is currently undergoing phase I clinical trials (Pegg et al, 1993; Hickson et al, 1996).

The effectiveness of the clinically used $O^6$-alkylating agents is limited by their relatively unselective and frequently dose-limiting toxicity to normal tissues. In response to this problem a number of molecular combinations of 5-fluorouracil (5FU) and chloroethylnitrosourea (CNU) have been developed (McElhinney et al, 1989*a*, 1989*b*) with the aim of producing agents with good activity against tumours but which may be less toxic to normal tissue. It was anticipated that the molecular combinations would result in a more gradual release in vivo of an active component, in a manner not possible when the two free components are administered separately. Studies to date on a limited number of these compounds have suggested that 5FU release per se does not seem to account for their anti-tumour activity, but instead indicates that the alkylating components of these drugs are highly effective in this conformation. Three of these compounds (Figure 1) were selected for further study on the basis of previously described preliminary anti-tumour studies: B.3995 (McElhinney et al, 1989*a*), in which the

*Received 6 March 1998*
*Revised 16 September 1998*
*Accepted 22 September 1998*

*Correspondence to*: MC Bibby

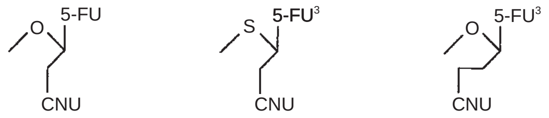

B.3995 B.3996 B.4152

**Figure 1** Molecular structures of the molecular combinations of CNU and 5FU. $5FU^3$ is 5-fluorouracil-3-yl ($N^3$-linked)

linear pseudo-sugar fragment linking the CNU and the 5FU by the normal $N^1$ has been greatly simplified, the closely-related B.3996 (McElhinney et al, 1989*b*), containing sulphur in place of the normal sugar oxygen and an $N^3$ link to the pyrimidine ring and B.4152 (Loadman et al, 1996) which retains all four carbon atoms of the furanose skeleton, although lacking the usual sugar hydroxyl groups. Previous studies have shown B.4152 to be relatively marrow-sparing in a mouse in vivo model (Loadman et al, 1996).

Here we report the results of a study designed to determine the capacity of BG to potentiate the anti-tumour activity of B.3995, B.3996 and B.4152, in comparison to BCNU, and to investigate the extent to which these molecular combinations are sparing to normal tissues, particularly the bone marrow.

### MATERIALS AND METHODS

### Animals

Pure strain NMRI mice from an inbred colony were purchased from B&K Universal Ltd, Hull, UK. NCR/Nu mice were obtained from the National Cancer Institute, Frederick, Maryland, USA. They were fed with a pellet diet (CRM, Special Diets Services, Witham, Essex, UK) and water ad libitum. Nude mice were housed in isolation cabinets. All mice had reached a minimum age of 8 weeks before use. Animal experiments were conducted in compliance with an approved project licence (Home Office, London, UK).

### Test compounds

BCNU was obtained from Bristol-Myers Pharmaceuticals (UK). BG and the molecular combinations B.3995, B.3996 and B.4152 were synthesized by Dr JE McCormick, Trinity College, Dublin (McCoss et al, 1985; McElhinney et al, 1989*a*, 1989*b*; McMurray et al, 1994; Loadman et al, 1996). BG was solubilized in Cremophor/saline (1/10 v/v), BCNU in ethanol/saline (1/10) and B-series compounds in dimethylsulphoxide (DMSO)/arachis oil (1/10 v/v). All treatments were by single intraperitoneal (i.p.) injection.

### Tumour systems

Fragments (~ 1–2 $mm^3$) of murine colon adenocarcinoma MAC-26 were transplanted into the flank of groups of male NMRI mice by means of a trocar (Loadman et al, 1996). The MAC-26 tumour was selected because of its general resistance to nitrosoureas. MT-1 human breast cancer xenografts (Naundorf et al, 1992) were similarly transplanted into female NCR nude mice.

### ATase activity

Tumour, liver, bone marrow and small intestine samples were obtained from NMRI and NCR-Nu mice after either 15 (NMRI only) or 120 min (NMRI and NCR-Nu) post-treatment with 60 mg $kg^{-1}$ BG or after being treated with the solvent vehicle only. Marrow samples for NCR-Nu were pooled before ATase activity determination because of low protein levels. Prior to assay, the biopsies were frozen in liquid nitrogen and stored at – 80°C. The ATase assay was performed as described previously (Lee et al, 1991, 1992), and is based upon the transfer of [$^3$H]methyl groups from substrate DNA to ATase protein present in tissue extracts. Briefly, tissue extracts were prepared at 4°C by sonication in buffer I (50 mM Tris-HCl, 3 mM DL-dithiothreitol and 2 mM EDTA, pH 8.3) containing leupeptin (0.5 µl $ml^{-1}$) with 10 µl phenylmethylsulphonyl fluoride (87 mg $ml^{-1}$) added after sonication. For the assay, aliquots of the extract were incubated with [$^3$H] substrate DNA at 37°C for 30 min, after which the DNA was hydrolysed in 1 M perchloric acid at 75°C for 50 min. The samples were then centrifuged and washed in 1 M perchloric acid before resuspension in 10 mM sodium hydroxide and aqueous scintillation fluid (Ecoscint A; National Diagnostics). Protein was estimated using the Bradford method (Bradford, 1976) with bovine serum albumin used as the standard. ATase activity was expressed as femtomoles (fmol) of methyl groups transferred to protein per milligram of total protein in the extract.

### Chemotherapy

Tumour-bearing animals were allocated into groups (minimum 8) and treatment commenced when tumours were large enough to be accurately measured by calipers (~ 4 × 4 mm). Anti-tumour effects were assessed by twice-weekly two-dimensional caliper measurements of the tumours and their volumes were calculated from the formula $a^2 \times b/2$, where a is the smaller diameter and b the larger (Geran et al, 1972). Growth curves were constructed and comparisons made between treated and control groups: solvent control groups were also included. Efficacy was determined on the basis of the difference in time taken for control and treated tumours to double in volume.

### Normal tissue toxicity

Gastrointestinal and hepatic toxicity was assessed in non-tumour bearing BALB/c mice following treatment with BCNU alone (20 mg $kg^{-1}$) or in combination with BG (60 mg $kg^{-1}$ 2 h prior to BCNU). After 3 days the animals were killed by cervical dislocation and the tissues of interest removed. These were immediately fixed in Bouin's reagent for 24 h, dehydrated and embedded in paraffin wax for sectioning before staining with haematoxylin and eosin (H & E) and examination by light microscopy.

Acute bone marrow toxicity was assessed using a modified version of the spleen colony forming assay of Till and McCulloch (1961), similar to that used elsewhere (Patchen, 1995; Lord et al, 1996). Using a single time-point of 24 h post-treatment (Siemann and Beyers, 1993; Down et al, 1994; Siemann, 1996), marrow cells were obtained from both femora of pairs of treated or control mice and suspended in RPMI tissue culture medium. Cell suspensions were diluted so that a 0.2 ml aliquot contained an appropriate number of cells for each experimental group. Cell inocula of $5.0 \times 10^4$ to $7.5 \times 10^5$ were initially obtained from marrows of control mice in order to investigate the relationship between the number of spleen colonies formed and the number of marrow cells inoculated intravenously (i.v.) via the tail vein. On the basis of these initial experiments cell inocula of 1.0 to $2.5 \times 10^5$ were used for further studies. Cells from control or treated mice were injected i.v. into recipient mice which had been exposed to a marrow ablative dose of X-irradiation (11.7 Gy) from a Newton Victor Superficial Therapy Unit (GX 10). Groups of six mice were used for each experimental point. After 8 days the mice were sacrificed, the spleens removed and fixed in Bouin's reagent and the nodules on the surface were counted. The surviving fraction was determined by comparison of the mean number of colonies observed with the number of colonies expected for a given cell inoculum of marrow cells from untreated mice.

### RESULTS

### ATase activity in NMRI mice and MAC-26 tumours

The mean ATase activity in the livers of NMRI mice was 121 ± 26 fmol mg$^{-1}$ total cellular protein. Fifteen minutes after treatment with 60 mg kg$^{-1}$ BG, this activity had rapidly decreased to below 2 fmol mg$^{-1}$ (i.e. less than 2% of the pretreatment levels), the limit of detection of this assay and substrate: 2 h after treatment the activity remained depressed at < 2 fmol mg$^{-1}$. The NMRI bone marrow had a control activity of 61 fmol mg$^{-1}$ which, after 2 h, had fallen to 17 fmol mg$^{-1}$ (28% of the pretreatment activity). The MAC-26 tumour had a mean control ATase activity of 84 ± 7 fmol mg$^{-1}$. Fifteen minutes after treatment with BG, the activity had fallen to 2 ± 0.2 fmol mg$^{-1}$ (2.5% of the pretreatment levels) and again, after 2 h activity was found to be below the level of detection of the assay.

### ATase activity in NCR-Nu mice and MT-1 tumours

Control NCR-Nu liver had a mean ATase activity of 55 ± 4 fmol mg$^{-1}$. Two hours after treatment with BG, this was reduced to < 2 fmol mg$^{-1}$ (i.e. to below 3.6% of the pretreatment level). The NCR-Nu marrow had a control activity of 30 fmol mg$^{-1}$ which, similarly to the NMRI marrow, was reduced to only 9 fmol mg$^{-1}$ (30% of the control ATase activity) 2 h after treatment with 60 mg kg$^{-1}$ BG. The MT-1 tumour had a mean activity of 262 ± 7 fmol mg$^{-1}$ which, 2 h after 60 mg kg$^{-1}$ BG, had been reduced to 4 ± 2 fmol mg$^{-1}$ (1.5% of the control).

When the assay was carried out on intestinal tissue, activity was not detected in any of the samples (treated or control), probably due to excess protease, released during the extraction procedure, reducing the ATase activity to below detectable levels.

### Anti-tumour studies

#### *Activity against MAC-26 tumours*

The results of anti-tumour studies using the experimental agents either alone or in combination with BG on MAC-26 xenografts are presented in Table 1. Growth delays analysed by a Mann-Whitney *U*-Test indicated significant ($P < 0.05$) anti-tumour activity for B.3995, B.3996 and B.4152 at their maximum tolerated doses (MTD) compared with BCNU alone which was not effective against this tumour even at the highest dose used. Combination with BG increased the normal tissue toxicity of each compound, requiring a reduction in their dose levels to ensure that equi-toxic drug levels were administered. In each case pretreatment with BG resulted in significant enhancement of the anti-tumour activities of the B-series compounds: this was particularly evident for B.3996 where effects were seen with a dose of only 15 mg kg$^{-1}$ combined with BG. Furthermore, even the lowest dose of BCNU administered was able to effect significant tumour growth delay if preceded by treatment with 60 mg kg$^{-1}$ BG. Statistical analysis of the tumour growth delay of the four different anti-tumour agents demonstrates that there is no significant difference ($P > 0.05$) between BCNU and the three B-series compounds when used in combination with BG.

#### *Activity against MT-1 tumours*

Anti-tumour studies with the human breast MT-1 xenograft model were not completed due to the excessive normal tissue toxicity observed when the treatments were combined with BG. Two dose levels of BCNU (20 and 10 mg kg$^{-1}$) were examined in combination with BG, neither of which when used alone were found to produce a significant growth delay. In combination, both doses produced anti-tumour effects but excessive weight loss resulted in the experiments being terminated to avoid suffering. However, the available data suggested that BG potentiated the anti-tumour activity of BCNU against MT-1, given that the relative tumour volumes of the BCNU/BG treated animals were 34% (10 mg kg$^{-1}$) and 50% (20 mg kg$^{-1}$) less than both the control or BCNU-only treated groups.

### Normal tissue toxicity

#### *Bone marrow*

The toxic effect of BCNU, either alone or in combination with BG, on the bone marrow spleen colony forming cells is presented in Table 2. Treatment with BG alone had no obvious effect on spleen colony formation, whereas BCNU had severe effects at doses of 40 and 20 mg kg$^{-1}$, reducing CFU-S survival to 3% and 21% respectively. A more modest toxicity was produced by a single dose of 10 mg kg$^{-1}$, with 77% of CFU-S surviving BCNU exposure.

**Table 1** Anti-tumour activity against MAC-26 murine colon adenocarcinoma.

| Treatment | Dose (mg kg$^{-1}$) | Growth delay (days) | Statistical significance |
|---|---|---|---|
| BG | 60 | 0 | – |
| BCNU | 40 | 0.20 | NS |
| BCNU | 20 | 0.05 | NS |
| BG/BCNU | 60/20 | 2.45 | $P < 0.05$ |
| B.3995 | 50* | 3.65 | $P < 0.05$ |
| B.3995 | 20 | 0.45 | NS |
| BG/B.3995 | 60/20 | 5.15 | $P < 0.01$ |
| B.3996 | 100* | 9.15 | $P < 0.01$ |
| B.3996 | 10 | 0.32 | NS |
| B.3996 | 15 | 1.12 | NS |
| BG/B.3996 | 60/10 | 1.60 | $P < 0.05$ |
| BG/B.3996 | 60/15 | 10.77 | $P < 0.01$ |
| B.4152 | 70* | 2.0 | $P < 0.05$ |
| B.4152 | 20 | –0.9 | NS |
| BG/B.4152 | 60/20 | 11.75 | $P < 0.01$ |

*Signifies maximum tolerated doses of single agents. In each combination experiment cytotoxic therapy was administered as a single i.p. dose 2 h after BG.

 © Cancer Research Campaign 1999

**Table 2** Influence of BG on the toxicity of BCNU to murine CFU-S

| Treatment | Dose (mg $kg^{-1}$) | Number of colonies observed* | Number of colonies expected | Survival fraction |
|---|---|---|---|---|
| BG | 60 | 20.7 ± 1.5 | 24.9 | 0.83 |
| BCNU | 40 | 0.83 ± 0.75 | 28.5 | 0.03 |
| BCNU | 20 | 5.0 ± 2.1 | 23.4 | 0.21 |
| BCNU | 10 | 18.5 ± 3.4 | 24.1 | 0.77 |
| BG/BCNU | 60/10 | 0 | 27.4 | 0 |

*Mean ± 1 SD

However, the toxicity of this dose was dramatically potentiated by BG, with no discernible colonies being present in animals exposed to the combination treatment.

Data from similar studies with B.3995, B.3996 and B.4152 are recorded in Table 3. In each case the molecular combinations caused a reduction in CFU-S at maximum tolerated dose and reduction in dose resulted in a corresponding reduction in bone marrow toxicity. Combination with BG at doses optimized for an anti-tumour effect resulted in very severe toxicity to the bone marrow with no CFU-S detected under these conditions.

### *Liver*

Liver toxicity was observed in animals treated with BG followed 2 h later by 20 mg $kg^{-1}$ BCNU, and consisted of numerous foci of intracellular fatty globulation (Figure 2A and B). Animals treated with either compound alone did not exhibit any observable toxicity.

### *Gastrointestinal tract*

The combination of BG with 20 mg $kg^{-1}$ BCNU produced a clear and dramatic change in the appearance of the intestine. The villi were lost from the small intestine with only a small amount of cellular debris remaining in the lumen (Figure 2C and D). Intestinal toxicity was not observed in the animals treated with BCNU or BG alone, with the villi and associated tissue appearing normal in morphology.

Body weight loss occurred when BG was combined with either BCNU or any of the B-series. Weight loss was most marked in the NCR/Nu and BALB/c mice where experiments had to be ended 4 days post-treatment due to excessive weight loss [NCR/Nu mean loss 25.7% (± 3.28) 4 days after 60 mg $kg^{-1}$ BG and 20 mg $kg^{-1}$ BCNU]. A reduction in the dose of BCNU to 10 mg $kg^{-1}$ resulted in reduced, but still excessive, weight loss (mean 16.16%, ± 0.04) after 3 days.

Weight loss was also observed in the NMRI mice, but to a lesser extent, reaching a nadir 7 days post-treatment with 60 mg $kg^{-1}$ BG and 20 mg $kg^{-1}$ BCNU (mean loss 6.43%, ± 2.79), increasing thereafter. When combined with BG, the B-series compounds produced some weight loss in the NMRI mice, but similarly to BCNU/BG, this was found to be 4–8% with a nadir of 7–8 days post-treatment, increasing after this point.

### DISCUSSION

The ATase inactivator BG has been widely reported to potentiate the anti-tumour activity of various established $O^6$-alkylating agents such as BCNU, CCNU and temozolomide (Dolan et al, 1990; Mitchell et al, 1992; Chinnasamy et al, 1997; Kurpad et al, 1997). Here we report the effect of several novel nitrosoureas, either alone or in combination with BG, on a murine colon adenocarcinoma (MAC-26) and a human breast carcinoma (MT-1).

**Table 3** Influence of BG on the toxicity of B.3995, B.3996 and B.4152 to murine CFU-S

| Treatment | Dose (mg $kg^{-1}$) | Number of colonies observed* | Number of colonies expected | Survival fraction |
|---|---|---|---|---|
| B.3995 | 50 | 5.5 ± 2.3 | 39.6 | 0.14 |
| B.3995 | 25 | 13.5 ± 1.1 | 31.4 | 0.43 |
| B.3995 | 20 | 12.0 ± 1.3 | 28.2 | 0.43 |
| BG/B.3995 | 60/20 | 0 | 57.5 | 0 |
| B.3996 | 100 | 2.8 ± 0.8 | 33.5 | 0.08 |
| B.3996 | 50 | 6.8 ± 0.8 | 40.4 | 0.17 |
| B.3996 | 25 | 18.2 ± 2.0 | 34.3 | 0.53 |
| B.3996 | 15 | 11.3 ± 1.5 | 22.9 | 0.49 |
| BG/B.3996 | 60/15 | 0 | 26.9 | 0 |
| B.4152 | 100 | 10 ± 1.1 | 31.25 | 0.32 |
| B.4152 | 50 | 12.2 ± 1.3 | 20 | 0.61 |
| B.4152 | 25 | 16.3 ± 1.6 | 22.32 | 0.73 |
| B.4152 | 20 | 20.8 ± 1.2 | 25.2 | 0.83 |
| BG/B.4152 | 60/20 | 0 | 36.6 | 0 |

*Mean ± 1 SD

Both tumour types express relatively high levels of ATase: the mean activities determined for MAC-26 and MT-1 were 84 ± 7 fmol $mg^{-1}$ and 262 ± 7 fmol $mg^{-1}$ respectively. These activities are similar to those found in other tumours such as human glioma xenografts (Kurpad et al, 1997), testis and bladder tumour cell lines (Walker et al, 1992) and human β-lymphoblastoid cell lines (Bronstein et al, 1992).

Prior to the anti-tumour and normal cell toxicity studies, the degree and kinetics of ATase depletion in the tissues to be analysed was determined. BG rapidly depleted the ATase activity in normal tissues of both mouse strains and also in both tumour xenografts. The speed and degree of the depletion in ATase activity is similar to that reported elsewhere for both normal (Dolan et al, 1990; Chinnasamy et al, 1997) and tumour tissue (Mitchell et al, 1992; Pegg at al, 1993), with a rapid and considerable depletion observed over the initial 15 min after the dose of BG. The two normal tissues examined responded slightly differently to the BG, with the liver ATase activity being depleted to below detectable levels, while the bone marrow maintained a higher ATase level after BG, confirming the observations reported by Chinnasamy et al (1997). The reasons for this tissue maintaining ATase activity are unclear, but it could be that non-haematopoietic cells in the marrow retain residual activity, or possibly differential penetration of the inactivator into different areas of the bone marrow occurs.

The level of ATase activity observed in the xenografts was apparently sufficient to protect against the anti-tumour effects of various doses of BCNU. However, it was possible to produce a significant growth delay in vivo against both tumour types, by pretreatment with the ATase-depleting doses of BG.

In view of the extreme sensitivity of NCR/nu mice to BG and treatment with BCNU, as evidenced by the excessive weight loss experienced by the mice during this regimen, the 5FU:CNU molecular combinations were evaluated in the syngeneic NMRI/MAC-26 murine model. These molecular combinations were designed to provide new agents offering effectiveness against previously problematical solid tumours. For all three investigational compounds,

© Cancer Research Campaign 1999

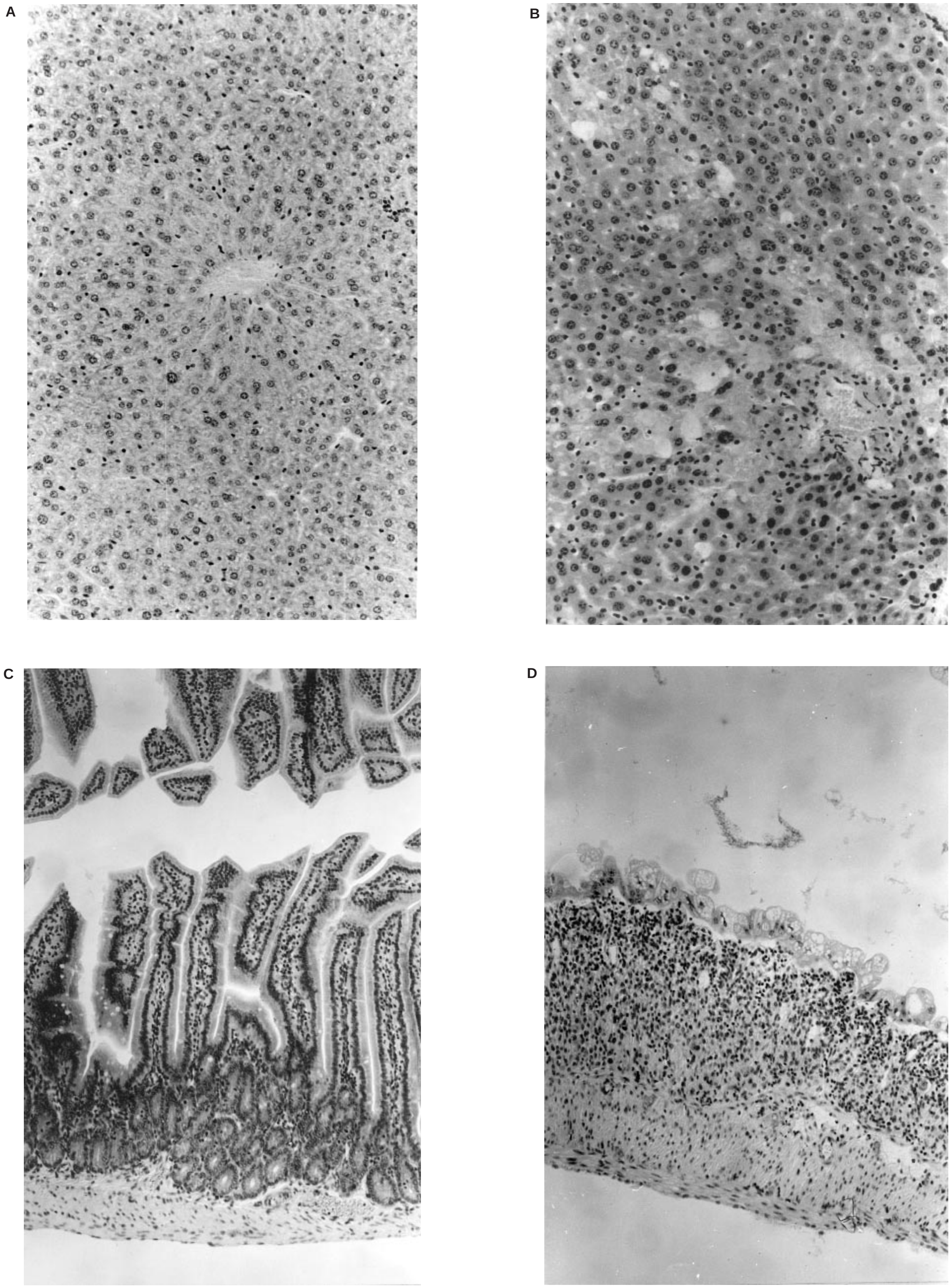

**Figure 2** Normal tissue toxicity after 60 mg kg$^{-1}$ BG and 20 mg kg$^{-1}$ BCNU. (**A**) Normal liver. (**B**) Liver with fatty globulation. (**C**) Normal small intestine. (**D**) loss of villi after treatment. H and E stained × 700

 *© Cancer Research Campaign 1999*

when compared to BCNU, significant growth delays were evident against MAC-26 tumours when the agents were used at their MTD. This growth delay was very significantly potentiated by pretreatment with BG: in particular, B.3996 and B.4152 were found to be about 4 and 5 times better respectively at inducing growth delay, if administered in combination with BG rather than alone at their MTD.

One of the important aims in designing the novel combinations was to attempt to reduce normal tissue toxicity, particularly towards the bone marrow. It is important in this regard to note that B.4152 as a single agent, while being significantly active against refractory mouse adenocarcinoma tumour models has also been shown to exert reduced myelotoxicity (Loadman et al, 1996). To determine the acute haematological toxicity of the novel agents, either alone or in combination with BG, the spleen colony forming unit assay was used as with other published work (Bibby et al, 1988, 1993; Hendricks et al, 1993; Matthew et al, 1993, 1994; Patchen, 1995; Loadman et al, 1996). In order to allow comparison with a clinically relevant agent, BCNU was also included in this study. In all cases, pretreatment with BG resulted in complete ablation of the CFU-S compartment, and thus in this assay of acute haematological toxicity, the novel nitrosoureas examined appear to offer no significant therapeutic advantage over BCNU when used in combination with BG. When used as single agents at their MTD, however, B.4152 again appears to be significantly more marrow-sparing than the two other B-series compounds, which in turn appear more sparing than BCNU.

Interestingly, the dose of BG used did not entirely deplete the ATase activity of bone marrow and approximately 30% activity (17 fmol $mg^{-1}$) remained at 2 h post-treatment, a situation similar to that observed by Chinnasamy et al (1997). This level of activity might be anticipated to confer some protection against nitrosourea toxicity. However, the lack of any colonies in the CFU-S assay suggest that the residual ATase activity observed in the marrow after BG treatment may originate from non-haematopoietic cells.

In the same animals, a severe potentiation of toxicity was also observed in both liver and intestine, as judged by histological examination, following a BG/BCNU regimen. We have previously reported that the testicular toxicity of nitrosoureas in mice is elevated as a result of BG exposure (Thompson et al, 1996). Thus, the widespread and non-tissue specific BG-mediated depletion of ATase may prove to be problematic when this agent is used in a clinical context.

What selectivity the novel 5FU:CNU molecular combinations possess appears to be totally abolished when the repair capacity for $O^6$-alkG is depleted or, as in the case of bone marrow, reduced. It seems more probable that these novel agents will be more useful as single agents, given their reduced toxicity towards normal tissues in this context. It is worthwhile to note that when compared to BCNU, the B-series compounds were found to be significantly more active when used as single agents and all produced reduced acute haematological toxicity.

Other alkylating agents are currently being investigated in combination with BG to determine whether they offer any advantage over BCNU. For example, the methylating agent temozolomide has shown promising activity in phase I and II clinical trials (Newlands et al, 1992; O'Reilly et al, 1993) against glioblastoma. Preclinical work has shown that in combination with BG, it may offer a therapeutic advantage over BCNU (Wedge et al, 1996; Wedge and Newlands, 1996), although dose and time of administration may be critical as other reports show enhanced haemopoietic toxicity of temozolomide/BG combinations (Fairbairn et al, 1995; Chinnasamy et al, 1997).

If BG is to be widely useful clinically, it may be necessary to attempt to select as targets for cytotoxic therapy those tumours which more rapidly accumulate BG than normal tissues (Kurpad et al, 1997) or to promote the selective uptake of BG into tumour tissues by exploiting differences between normal tissues and the relatively abnormal physiology and microenvironment of tumours. Alternatively, it may be possible to protect haematopoietic cells in vivo from the biological effects of $O^6$-alkylating agents while simultaneously allowing the sensitization of tumours to these agents using BG (reviewed in Rafferty et al, 1996). Mutations in the human ATase that lead to resistance to BG inactivation have been reported (Crone and Pegg, 1993) and expression of these cDNAs in cell lines has been shown to provide resistance to the cytotoxic effects of combined BG and nitrosourea treatment (Hickson et al, 1996; Loktionova and Pegg, 1996). Furthermore, retroviral transduction of human primary haemopoietic progenitors ($CD34^+$) with cDNAs encoding BG resistant mutants of ATase also resulted in increased resistance to BG with either BCNU (Reese et al, 1996) or temozolomide (Hickson et al, 1997), encouraging clinical trials of such strategies.

It is always difficult to predict clinical outcome from murine studies and the ultimate test of the value of BG-mediated tumour sensitization approaches will emerge from ongoing clinical trials. However, it is hoped that a generation of more tumour-selective ATase inactivators and anti-tumour agents, perhaps used in conjunction with gene therapy protection strategies, will define a more successful approach.

### ACKNOWLEDGEMENTS

We would like to thank Dr Anne Matthew for help with the in vivo chemotherapy and bone marrow CFU-S assays. The work was supported by the War on Cancer and Cancer Research Campaigns.

### REFERENCES

Baer JC, Freeman AA, Newlands ES, Watson AJ, Rafferty JA and Margison GP (1993) Depletion of $O^6$-alkylguanine-DNA-alkyltransferase correlates with potentiation of temozolomide in human tumour cells. *Br J Cancer* **67**: 1299–1302

Bibby MC, Double JA, Wahed IA, Hirbawi (Abu-Khalaf) N and Baker TG (1988) The logistics of broader pre-clinical evaluation of potential anti-cancer agents with reference to anti-tumour activity and toxicity of mitozolomide. *Br J Cancer* **58**: 139–143

Bibby MC, Sleigh NR, Loadman PM and Double JA (1993) Potentiation of EO-9 anti-tumour activity by hydralazine. *Eur J Cancer* **29A**: 897–906

Bradford M (1976) A rapid and sensitive method for quantitation of microgram quantities of protein utilising the principle of protein-dye binding. *Anal Biochem* **72**: 248–254

Brennand J and Margison GP (1986) Reduction of the toxicity and mutagenicity of alkylating agents in mammalian cells harbouring the *Escherichia coli* alkyltransferase gene. *Proc Natl Acad Sci USA* **83**: 6292–6296

Brent TP, von Wronski MA, Edwards CC, Bromley M, Margison GP, Rafferty JA, Pegram CN and Bigner DD (1993) Identification of nitrosourea-resistant human rhabdomyosarcomas by *in situ* immunostaining of $O^6$-methylguanine-DNA-methyltransferase. *Oncol Res* **5**: 83–86

Bronstein SM, Hooth MJ, Swenberg JA and Skopek TR (1992) Modulation of ethylnitrosourea-induced toxicity and mutagenicity in human cells by $O^6$-benzylguanine. *Cancer Res* **52**: 3851–3856

Chinnasamy N, Rafferty JA, Hickson I, Ashby J, Tinwell H, Margison G, Dexter TM and Fairbairn LJ (1997) $O^6$-benzylguanine potentiates the in vivo toxicity

© Cancer Research Campaign 1999

and clastogenicity of temozolomide and BCNU in mouse bone marrow. *Blood*, **89**: 1566–1573

Crone TM and Pegg AE (1993) A single amino acid change in human *O*6-alkylguanine-DNA alkyltransferase decreasing sensitivity to inactivation by *O*6-benzylguanine. *Cancer Res* **53**: 4750–4753

Dolan ME, Chae MY, Pegg AE, Mullen JH, Friedman HS and Moschel RC (1994) Metabolism of *O*6-benzylguanine, an inactivator of *O*6-alkylguanine-DNA alkyltransferase. *Cancer Res* **54**: 5123–5130

Dolan ME, Corsico CD and Pegg AE (1985) Exposure of HELA cells to *O*6-alkylguanines increases the sensitivity to the cytotoxic effects of alkylating agents. *Biochem Biophys Res Commun* **132**: 178–185

Dolan EM, Moschell RC and Pegg AE (1990) Depletion of mammalian *O*6-alkylguanine-DNA-alkyltransferase activity by *O*6-benzylguanine provides a means to evaluate the role of this protein in protection against carcinogenic and therapeutic alkylating agents. *Proc Natl Acad Sci USA* **87**: 5368–5372

Dolan ME, Stine L, Mitchell RB, Moschel RC and Pegg AE (1990) Modulation of mammalian *O*6-alkylguanine-DNA alkyltransferase *in vivo* by *O*6-benzylguanine and its effect on the sensitivity of a human glioma tumour to 1-(2-chloroethyl)-3-(4-methylcyclohexyl)-1-nitrosourea. *Cancer Communications* **2**: 371–377

Dolan ME, Pegg AE (1997) *O*6-benzylguanine and its role in chemotherapy. *Clinical Cancer Research* **3**: 837–847

Down JD, Boudewijn A, Dillingh JH, Fox BW and Ploemacher RE (1994) Relationships between ablation of distinct haematopoietic cell subsets and the development of donor bone marrow engrafment following recipient pretreatment with different alkylating drugs. *Br J Cancer* **70**: 611–616

Dumenco LL, Allay E, Norton K and Gerson SL (1993) The prevention of thymic lymphomas in transgenic mice by human *O*6-alkylguanine-DNA alkyltransferase. *Science* **259**: 219–222

Fairbairn LJ, Watson AJ, Rafferty JA, Elder RH and Margison GP (1995) *O*6-benzylguanine increases the sensitivity of human primary bone marrow cells to the cytotoxic effects of temozolomide. *Experimental Haematology* **23**: 112–116

Geran RI, Greenberg NH, MacDonald MM, Schumacher AM and Abbott BJ (1972) Protocols for screening chemical agents and natural products against animal tumours and other biological systems (third edition). *Cancer Chemother Report*s **3**: 1–103

Gerson SL, Trey JE and Miller K (1988) Potentiation of nitrosourea cytotoxicity in human leukemic cells by inactivation of *O*6-alkylguanine-DNA alkyltransferase. *Cancer Res* **48**: 1521–1527

Hendriks HR, Piazo PE, Berger DP, Kooistra KL, Bibby MC, Boven E, Dreefvandermeulen HC, Henrar REC, Fiebeg HH, Double JA, Hornstra HW, Pinedo HM, Workman P and Schwartsmann G (1993) EO9 – a novel bioreductive alkylating indolquinone with preferential solid tumour activity and lack of bone marrow toxicity in preclinical models. *Eur J Cancer* **29A**: 897–906

Hickson I, Fairbairn LJ, Chinnasamy N, Margison GP, Dexter TM and Rafferty JA (1996) Protection of mammalian cells against chloroethylating agent toxicity by an *O*6-benzylguanine-resistant mutant of human *O*6-alkylguanine-DNA-alkyltransferase. *Gene Ther* **3**: 868–877

Hickson I, Fairbairn LJ, Chinnasamy N, Dexter TM, Margison GP and Rafferty JA (1997) Towards gene therapy for stem cell protection using mutant human *O*6-alkylguanine-DNA alkyltransferase. *Cancer Gene Ther* **4**: 70

Jelinek J, Kleibl K, Dexter TM and Margison GP (1988) Transfection of murine multi-potent haemopoietic stem cells with an *E. coli* DNA alkyltransferase gene confers resistance to the toxic effects of alkylating agents. *Carcinogenesis* **9**: 81–87

Jelinek J, Fairbairn LJ, Dexter TM, Rafferty JA, Stocking C, Ostertag W and Margison GP (1996) Long-term protection of haemopoiesis against the cytotoxic effects of multiple doses of nitrosourea by retrovirus-mediated expression of human *O*6-alkylguanine-DNA-alkyltransferase. *Blood* **87**: 957–1961

Kaina B, Fritz G, Mitra S and Coquerelle T (1991) Transfection and expression of human *O*6-methylguanine DNA-methyltransferase (MGMT) cDNA in Chinese hamster cells: the role of MGMT in protection against the genotoxic effects of alkylating agents. *Carcinogenesis* **11**: 777–780

Kurpad SN, Dolan EM, McLendon RE, Archer GE, Moschel RC, Pegg AE, Bigner DD and Friedman HS (1997) Intra-arterial *O*6-benzylguanine enables the specific therapy of nitrosourea-resistant intracranial human glioma xenografts in athymic rats with 1,3-bis(2-chloroethyl)-1-nitrosourea. *Cancer Chemother Pharmacol* **39**: 307–316

Lee SM, Thatcher N and Margison GP (1991) *O*6-alkylguanine-DNA-alkyltransferase depletion and regeneration in human peripheral lymphocytes following dacarbazine and fotemustine. *Cancer Res* **51**: 619–623

Lee SM, Thatcher N, Crowther D and Margison GP (1992) In vivo depletion of *O*6-alkylguanine-DNA-alkyltransferase in lymphocytes and melanoma of patients treated with CB10-277, a new DTIC analogue. *Cancer Chemother Pharmacol* **31**: 240–246

Loadman PM, Matthew AM, McCormick JE, McElhinney RS and Bibby MC (1996) Pharmacokinetics and antitumour activity of B.4152, a reactive molecular combination of 5-fluorouracil and N-(2chloroethyl)-N-nitrosourea, and its uracil analogue. *Anticancer Drug Des* **11**: 117–128

Loktionova NA and Pegg AE (1996) Point mutations in human *O*6-alkylguanine-DNA-alkyltransferase prevent the sensitisation by *O*6-benzylguanine to killing by *N,N′*-bis(2-chloroethyl)-N-nitrosourea. *Cancer Res* **56**: 1578–1583

Lord BI, Marshall E, Woolford LB and Hunter MG (1996) BB-10010/MIP-1α in vivo maintains haemopoietic recovery following repeated cycles of sublethal irradiation. *Br J Cancer* **74**: 1017–1022

Margison GP, Hickson I, Jelinek KJ, Elder RH, Rafferty JA, Faibairn LJ, Dexter TM, Stocking C, Baum C, Ostertag W, Donnelly D, McMurray TBH, McCormick J and McElhinney RS (1996) Resistance to alkylating agents: more or less. *Anticancer Drugs* **7**: 109–116

Margison GP and O'Connor PJ (1990) Biological consequences of reactions with DNA: role of specific lesions. In *Handbook of Experimental Pharmacology 94/1*, Cooper CS and Grover PL (eds) pp 547–571. Springer-Verlag: Berlin

Matthew AM, Phillips RM, Loadman PM and Bibby MC (1993) Preclinical evaluation of a novel chloroethylating agent, clomesome. *Br J Cancer* **67**: 441–446

Matthew AM, Christensson PI, Seidegard J, Tuvesson H, Bibby MC, Hartleyasp B and Craven PJ (1994) Effect of drug scheduling on the anti-tumour activity and bone marrow toxicity of tauromustine (TCNU). *International Journal of Oncology* **4**: 403–409

McCoss M, Chen A and Tolman RL (1998) Synthesis of the chiral acyclonucleoside antiherpetic agent (S)-9-(2,3-dihydroxy-1-propoxymethyl)guanine. *Tetrahedron Letters* **26**: 1815–1818

McElhinney RS, McCormick JE, Bibby MC, Double JA, Atassi G, Dumont P, Pratesi G and Radacic M (1989a) Nucleoside analogues: 7. Effects on colon, breast and lung tumours in mice of 5-fluorouracil/nitrosourea combinations incorporating aloxy and oxidised sulphur fractions. *Anticancer Drugs* **3**: 255–269

McElhinney RS, McCormick JE, Bibby MC, Double MC, Atassi G, Dumont P, Pratesi G and Radacic M (1989b) Nucleoside analogues: 8. Some isomers of B.3839, the original 5-fluorouracil/nitrosourea molecular combination, and their effect on colon, breast and lung tumours in mice. *Anticancer Drug Des* **4**: 1–20

McMurray TBH, McElhinney RS, McCormick JE, Elder RH, Kelly J, Margison GP, Rafferty JA, Watson AJ and Willington MA (1994) Preparation of *O*6-substituted guanine derivatives as anti-tumour agents. *International Patent Application WO94/29, 312*, 22 December

Mitchell RB, Moschel RC and Dolan ME (1992) Effect of *O*6-benzylguanine on the sensitivity of human tumour xenografts to 1,3-bis(2-chloroethyl)-1-nitrosourea and on DNA interstrand cross-link formation. *Cancer Res* **52**: 1171–1175

Moritz T, Mackay W, Glassner BG, Williams DA and Samson L (1995) Retrovirus-mediated expression of a cDNA repair protein in bone marrow protects haematopoietic cells from nitrosourea-induced toxicity in vitro and in vivo. *Cancer Res* **55**: 2608–2614

Nakatsuru Y, Matsukuma S, Nemoto N, Sugano H, Sekiguchi M and Ishikawa T (1993) *O*6-methylguanine-DNA methyltransferase protects against nitrosamine-induced hepatocarcinogenesis. *Proc Natl Acad Sci USA* **90**: 6468–6472

Naundorf H, Rewasowa EC, Fichtner I, Buttner B, Becker M & Gorlish M (1992) Characterisation of two human primary mammary carcinomas, MT-1 and MT-3, suitable for in vivo testing of ether lipids and their derivatives. *Breast Cancer Res Treat* **23**: 87–95

Newlands ES, Blackledge GRP, Slack JA, Rustin GJS, Smith DB, Stuart NSA, Quarterman CP, Hoffman R, Stevens MFG, Brampton MH and Gibson AC (1992) Phase I trial of temozolomide (CCRG 81045: M and B 39831 NSC 362856). *Br J Cancer* **65**: 287–291

O'Reilly SM, Newlands ES, Glaser MG, Brampton M, Rice-Edwards JM, Illingworth RD, Richards PG, Kennard C, Colquhoun IR, Lewis P and Stevens MFG (1993) Temozolomide: a new oral cytotoxic chemotherapeutic agent with promising activity against primary brain tumours. *Eur J Cancer* **29A**: 940–942

Patchen ML (1995) Amifostine plus granulocyte colony-stimulating factor therapy enhances recovery from supralethal radiation exposures: preclinical experience in animal models. *Eur J Cancer* **31A, S1**: S17–S21

Pegg AE and Dolan ME (1987) Properties and assay of mammalian *O*6-alkylguanine-DNA alkyltransferase. *Pharmacol Ther* **34**: 167–179

*© Cancer Research Campaign 1999*

Pegg AE (1990) Mammalian $O^6$-alkylguanine-DNA alkyltransferase: regulation and importance in response to alkylating carcinogenesis and therapeutic agents. *Cancer Res* **50**: 6119–6129

Pegg AE and Byers TL (1992) Repair of DNA containing $O^6$-alkylguanine. *FASEB J* **6**: 2302–2310

Pegg AE, Boosalis M, Samson L, Moschel RC, Byers TL, Swenn K and Dolan ME (1993) Mechanism of inactivation of human $O^6$-alkylguanine-DNA alkyltransferase by $O^6$-benzylguanine. *Biochemistry* **32**: 11998–12006

Rafferty JA, Hickson I, Chinnasamy N, Lashford LS, Margison GP, Dexter TM and Fairbairn LJ (1996) Chemoprotection of normal tissues by transfer of drug resistance genes. *Cancer Metastasis Rev* **15**: 365–383

Reese JS, Koc ON, Lee KM, Liu LL, Allay JA, Phillips WP and Gerson SL (1996) Retroviral transduction of mutant methylguanine DNA methyltransferase gene into human CD34 cells confers resistance to O6-benzylguanine plus 1,3-bis (2-chloroethyl)-1-nitrosourea. *Proc Natl Acad Sci USA* **93**: 14088–14093

Siemann DW (1996) The in situ tumour response to combinations of cyclophosphamide and tirpazamine. *Br J Cancer* **74 S27**: S65–S69

Sieman DW and Byers KL (1993) In vivo therapeutic potential of combination thiol depletion and alkylating chemotherapy. *Br J Cancer* **68**: 1071–1079

Thompson MJ, Abdul Rahman S, Baker TG and Bibby MC (1996) Potentiation of testicular cytotoxicity by the alkyltransferase inhibitor $O^6$-benzylguanine and the 5-fluorouracil/N-(2-chloroethyl)-N-nitrosourea molecular combination B.4152. *Reprod Toxicol* **10**: 71–77

Till JE and McCulloch EA (1961) A direct measurement of the radiation sensitivity of normal mouse bone marrow cells. *Radiat Res* **14**: 213–222

von Hofe E, Fairbairn L and Margison GP (1992) Relationship between $O^6$-alkylguanine-DNA alkyltransferase activity and N-methyl-N′-nitro-N-nitrosoguanidine-induced mutation, transformation, and cytotoxicity in C3H/10T1/2 cells expressing exogenous alkyltransferase genes. *Proc Natl Acad Sci USA* **89**: 11199–11203

Walker MC, Masters JRW and Margison GP (1992) $O^6$-alkylguanine-DNA-alkyltransferase activity and nitrosourea sensitivity in human cancer cell lines. *Br J Cancer* **66**: 840–843

Wedge SR, Porteous JK, May BL and Newlands ES (1996) Potentiation of temozolomide and 1,3-bis(2-chloroethyl)-nitrosourea cytotoxicity of $O^6$-benzylguanine: a comparative study in vitro. *Br J Cancer* **73**: 482–490

Wedge SR and Newlands ES (1996) $O^6$-benzylguanine enhances the sensitivity of a glioma xenograft with low $O^6$-alkylguanine-DNA alkyltransferase activity to temozolomide and BCNU. *Br J Cancer* **73**: 1049–1052

Yarosh DB (1985) The role of $O^6$-methylguanine-DNA methyltransferase in cell survival, mutagenesis and carcinogenesis. *Mutat Res* **145**: 1–16

*© Cancer Research Campaign 1999*
